# *Plasmodium falciparum* drug resistance-associated mutations in isolates from children living in endemic areas of Burkina Faso

**DOI:** 10.1186/s12936-023-04645-9

**Published:** 2023-07-20

**Authors:** Casimire Wendlamita Tarama, Harouna Soré, Mafama Siribié, Siaka Débé, Réné Kinda, Adama Ganou, Wendyam Gérard Nonkani, Farida Tiendrebeogo, Winnie Bantango, Kassoum Yira, Aladari Sagnon, Sonia Ilboudo, Esther Yéri Hien, Moussa Wandaogo Guelbéogo, NFale Sagnon, Yves Traoré, Didier Ménard, Adama Gansané

**Affiliations:** 1 Centre National de Recherche et de Formation sur le paludisme, Ouagadougou, Burkina Faso; 2 Université Joseph KI-ZERBO, Ouagadougou, Burkina Faso; 3 Malaria Genetic and Resistance Unit, Institut Pasteur, Université Paris Cité, INSERM U1201, 75015 Paris, France; 4 Malaria Parasite Biology and Vaccines, Institut Pasteur, Université Paris Cité, 75015 Paris, France; 5 Institute of Parasitology and Tropical Diseases, Université de Strasbourg, UR7292 Dynamics of Host-Pathogen Interactions, 67000 Strasbourg, France; 6 Laboratory of Parasitology and Medical Mycology, CHU Strasbourg, 67000 Strasbourg, France

**Keywords:** Malaria, *Plasmodium falciparum*, ACT, Sulfadoxine-pyrimethamine, *Pfcrt*, *Pfmdr-1*, *Dhfr*, *Dhps*, Burkina Faso

## Abstract

**Background:**

Artemisinin-based combinations therapy (ACT) is the current frontline curative therapy for uncomplicated malaria in Burkina Faso. Sulfadoxine-pyrimethamine (SP) is used for the preventive treatment of pregnant women (IPTp), while SP plus amodiaquine (SP-AQ) is recommended for children under five in seasonal malaria chemoprevention (SMC). This study aimed to assess the proportions of mutations in the *P. falciparum multidrug-resistance 1* (*Pfmdr1*), *P. falciparum chloroquine resistance transporter (Pfcrt), P. falciparum dihydrofolate reductase (pfdhfr)*, and *P. falciparum dihydropteroate synthase (pfdhps)*, genes from isolates collected during household surveys in Burkina Faso.

**Methods:**

Dried blood spots from *Plasmodium falciparum*-positive cases at three sites (Orodara, Gaoua, and Banfora) collected during the peak of transmission were analysed for mutations in *Pfcrt* (codons 72–76, 93, 97, 145, 218, 343, 350 and 353), *Pfmdr-1* (codons 86, 184, 1034, 1042 and 1246) *dhfr* (codons 51, 59, 108, 164) and *dhps* (at codons 431, 436, 437, 540, 581, 613) genes using deep sequencing of multiplexed Polymerase chaine reaction (PCR) amplicons.

**Results:**

Of the 377 samples analysed, 346 (91.7%), 369 (97.9%), 368 (97.6%), and 374 (99.2%) were successfully sequenced for *Pfcrt, Pfmdr-1, dhfr*, and *dhps*, respectively. Most of the samples had a *Pfcrt* wild-type allele (89.3%). The 76T mutation was below 10%. The most frequent *Pfmdr-1* mutation was detected at codon 184 (Y > F, 30.9%). The single mutant genotype (N**F**SND) predominated (66.7%), followed by the wild-type genotype (NYSND, 30.4%). The highest *dhfr* mutations were observed at codon 59R (69.8%), followed by codons 51I (66.6%) and 108 N (14.7%). The double mutant genotype (AC**IR**SI) predominated (52.4%). For mutation in the *dhps* gene, the highest frequency was observed at codon 437 K (89.3%), followed by codons 436 A (61.2%), and 613 S (14.4%). The double mutant genotype (I**AK**KAA) and the single mutant genotype (IS**K**KAA) were predominant (37.7% and 37.2%, respectively). The most frequent *dhfr/dhps* haplotypes were the triple mutant AC**IR**SI/I**A**KKAA (23%), the wild-type ACNCSI/ISKKAA (19%) and the double mutant AC**IR**SI/ISKKAA (14%). A septuple mutant AC**IRN**I/**VAK**K**G**A was observed in 2 isolates from Gaoua (0.5%).

**Conclusion:**

The efficacy of ACT partner drugs and drugs used in IPTp and SMC does not appear to be affected by the low proportion of highly resistant mutants observed in this study. Continued monitoring, including molecular surveillance, is critical for decision-making on effective treatment policy in Burkina Faso.

## Background

There were an estimation of 247 million malaria cases and 619,000 deaths in 2021 worldwide [1]. The World Health Organization (WHO) African Region, with an estimated 234 million malaria cases, accounted for about 95% of global cases, and about 96% of deaths [1]. Approximately 76% of all malaria deaths in 2021 occurred in African children under the age of five [1]. In 2020, Burkina Faso was identified by the WHO as a “High Burden to High Impact” (HBHI) country. Despite efforts to accelerate the reduction of malaria incidence and mortality through prevention, diagnosis, and treatment, the decline in malaria incidence is slow. Malaria is still a threat to public health as it remains the first cause of consultation (37.2%) in Burkina Faso [2]. The incidence of malaria varies in the country according to the epidemiological facies and is higher in children under 5 years of age (1237‰) than in the general population (568‰) [2]. Malaria is permanent in the southern and south-western regions, seasonal with a long duration of 4–6 months in the centre and a short duration of 2–3 months in the north of the country. More than 12 million cases of malaria have been identified in Burkina Faso, among which 605,504 cases of severe malaria and 4355 deaths in 2021 [2].

Anti-malarial drugs are important components of malaria control and prevention programmes [3]. They are used in the prevention of the disease through chemoprophylaxis and in curative treatment to prevent the progression of severe disease [3]. Having an effective treatment for malaria is a key to achieve the goals set by the WHO through the Global Technical Strategy for Malaria 2016–2030. Artemisinin-based combination treatment associates short-acting artemisinin with a longer-acting partner drug to reduce the emergence of resistance [4].

In Burkina Faso, due to widespread resistance to chloroquine (CQ), dihydroartemisinin-piperaquine (DHA-PPQ), artesunate-pyronaridine (ASPYR) and artemether-lumefantrine (AL) are recommended as first-line treatment for uncomplicated malaria. Sulfadoxine-pyrimethamine (SP) is used for intermittent preventive treatment of malaria during pregnancy (IPTp) and SP plus amodiaquine (SP-AQ) for seasonal malaria chemoprevention in children under five (SMC) [5]. The emergence and spread of parasite resistance to artemisinin derivatives and partner drugs used in ACT threatens progress in the fight against malaria.

To date, although none of the validated mutations have been found in the propeller domain of the *Pfkelch13* gene associated with artemisinin partial resistance in Burkina Faso [6–8], the results of the TES undertaken in 2017–2018 showed insufficient efficacy of AL at day 28 and DHA-PPQ at day 42 [8]. Furthermore, the Greater Mekong Sub-region and several parts of Africa, including Rwanda, Uganda, and the Horn of Africa, have reported parasite resistance to artemisinin [9–11]. While resistance to artemisinin alone rarely leads to ACT treatment failure, resistance to both artemisinin and partner drugs in ACTs can lead to high rates of treatment failure, as seen recently in parts of the Greater Mekong Region [11–14]. The use of molecular markers for the monitoring of anti-malarial drug resistance is one of the most valuable methods for the surveillance of drug efficacy [15].

Anti-malarial drug resistance is caused by single nucleotide polymorphisms (SNPs) in several *P. falciparum* genes, such as *P. falciparum multidrug-resistance 1* (*Pfmdr1*), *P. falciparum chloroquine resistance transporter* (*Pfcrt*), *P. falciparum dihydrofolate reductase (pfdhfr*), and *P. falciparum dihydropteroate synthase* (*pfdhps*) [15–17].

The *Pfcrt* mutation at codon 76 (K – T) usually associated with other nonsynonymous mutations (at codons 72, 74, or 75) [18, 19] is the primary mediator of chloroquine resistance, by increasing the export of chloroquine from the food vacuole, away from its target [20]. For example, chloroquine resistance associated with mutations in the *Pfcrt* gene have long been described in malaria-endemic regions [17, 21]. In the Greater Mekong Subregion, newly identified *Pfcrt* mutations have been associated with resistance to piperaquine, another 4-aminoquinoline-based drug [22, 23]. *Pfmdr-1* gene encodes an ABC transporter (ATP-binding cassette, P-glycoprotein homolog). MDR1, located in the membrane of the food vacuole, is involved in the modulation of the susceptibility to several anti-malarial drugs and, more particularly, in the hydrophobic anti-malarial efflux [24]. A strong association has been observed between the polymorphism in *Pfmdr-1* at the N86Y, 184 F and D1246Y positions or the amplification of the gene and parasite in vitro susceptibility to mefloquine, quinine, and artemisinin derivatives [22]. *Pfdhfr* and *pfdhps* genes code for an enzyme involved in the folate synthesis pathway [16, 25]. Dihydrofolate reductase-thymidylate synthase (DHFR) and Dihydropteroate synthase (DHPS) are the target of antifolate drugs, such as pyrimethamine and sulfadoxine. The accumulation of several specific nonsynonymous mutations in the *pfdhfr* gene results in high clinical treatment failure rates and increased in vitro sensitivity to pyrimethamine [26]. Resistance to sulfadoxine and dapsone drugs, most commonly involves the changes at codons S436A, A437G, K540E, A581G, and A613S [25]. In addition, both mutations in the *dhps* (S436A, A437G, K540E, A581G, and A613S) and *dhfr* (N51I, C59R, S108N, and I164L) genes have been shown to confer resistance to SP, used alone for intermittent preventive treatment in pregnant women (IPTp) and in combination with amodiaquine for seasonal malaria chemoprevention (SMC) [25, 27].

This study aimed to investigate the frequency of mutations associated with resistance to different anti-malarial drugs in the *Pfcrt, Pfmdr-1, dhfr*, and *dhps* genes in a part of the country where malaria incidence remains very high despite multiple interventions implemented by the Burkina Faso National Malaria Control Programme.

## Methods

### Study sites

**Fig. 1:**
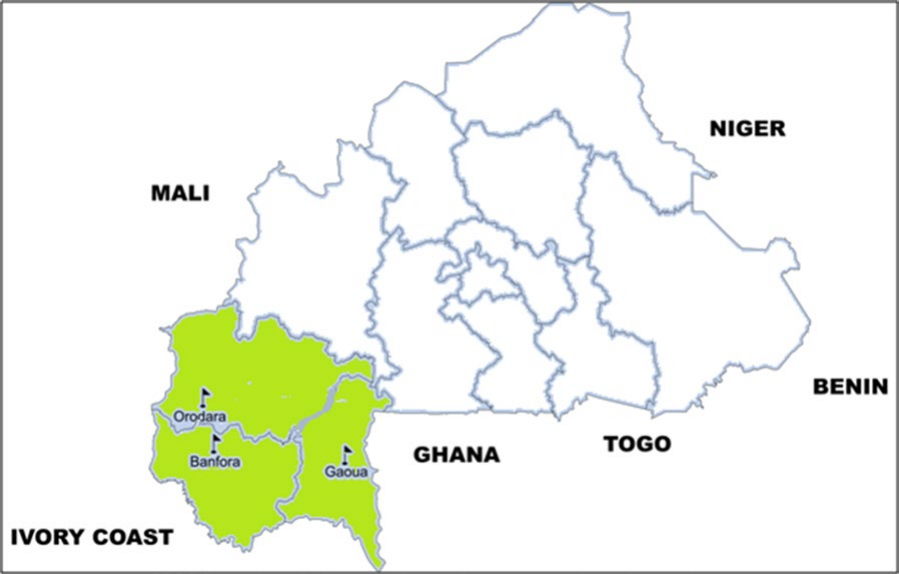
Study sites, Burkina Faso, 2022–2023

The study was conducted in the western and south-western regions, in the health districts of Banfora, Orodara, and Gaoua (Fig. 1). This part of the country is highly humid with rainfall ranging from 1000 to 1200 mm per year. Malaria is holoendemic with a peak of transmission from June to October. The main vectors of malaria are members of the *Anopheles gambiae* complex. The entomological inoculation rate (EIR) ranges from 0.91 to 2.35 infective bites/person/day [28, 29]. The Banfora, Orodara, and Gaoua health districts cover an area of 6266 km^2^, 8404 km^2^, and 7472 km^2^, respectively, with a population of 401,381, 259,974 and 251,611 [30]. Malaria prevalence and incidence in 2021 were 63.2% and 537.5‰, 57.1% and 541.9‰ and 77% and 860‰, respectively [2]. Coverage of seasonal chemoprophylaxis was estimated over 100% [2].

### Study design and period

The study was conducted through a cross-sectional household survey at the beginning of the rainy season in July 2022, the peak period for malaria transmission. Briefly, a minimum of 190 households with a child aged between 6 and 59 months and/or 190 children aged between 5 and 10 years in each study district were sampled using a standard systematic sampling approach, clustered by sector [31]. A total of 380 children were enrolled in 10 randomly selected villages in each health district. Households were selected if they involved children in the selected age range. Written informed consent was required from the parent or legal guardian of the child. The present study was part of a larger research project that aimed to investigate the prevalence of asymptomatic malaria using rapid diagnostic tests. Therefore, individuals with known malaria, undergoing anti-malarial treatment, or had received anti-malarial treatment within a month prior to their enrollment date were excluded from the study.

### Blood collection and malaria screening processing

Blood samples were taken aseptically from each participant by finger prick using a sterile disposable lancet by well-trained nurses who took the axillary temperature of selected children. A 50-µL drop of blood sample was then spotted on a 903TM Whatman TM (LASEC 10,530,151 Rev.AC, USA) five-spot blood card. Thick and thin blood films were made on the same slide and labeled with a unique code. The blood spots on the filter paper were individually air-dried and stored at room temperature in a sealed plastic bag containing a desiccant. All field samples (thick, thin, and dried spots) were then transported to the CNRFP laboratory in Ouagadougou, Burkina Faso for processing and analysis.

### Light microscopy screening

Thin blood smears were fixed in methanol for 30 s. The smears were then air-dried and stained with 3% Giemsa solution (QCA#990,939) for 45 min. Following the standard protocols of the CNRFP, the thick and thin blood smears were read by two independent level 1 certified malaria microscopists of the Clinical Laboratory Service. If the two readers disagreed on the species, presence, or absence of malaria parasites, or if the parasite densities differed by > 25%, the slide was re-read by a third independent microscopist. The mean of the two closest readings was used as the final value for parasite density. Parasites were counted on the thick film and the parasite density was calculated using the formula: number of parasites counted x 8000/number of leukocytes counted. A slide was considered negative if no parasites were detected in at least 200 fields.

### Parasite DNA extraction, PCR analysis, and DNA sequencing

The filter paper blood samples collected from an individual were stored in individual plastic bags with desiccant and protected from light, moisture, and extremes of temperature until they were analyzed. Each dried blood spot was sterile cut-out and placed in an Eppendorf tube. Parasite DNA was extracted under the protocol of Zainabadi et al. [32] in a 96-well format. Eluted DNA was then quantified by fluorometric quantification (Qubit, Thermo Fisher), adjusted to 20 ng/µL, and stored at − 20 °C for later use.

DNA was analysed for the presence of point mutations in *Pfcrt* (at codons 72–76, 93, 97, 145, 218, 343, 350, and 353), *Pfmdr-1* (at codons 86, 184, 1034, 1042, and 1246) genes associated or suspected with 4-aminoquinoline and amino alcohol resistance, *dhfr* (at codons 51, 59, 108, 164) and *dhps* (at codons 431, 436, 437, 540, 581, 613) genes associated with pyrimethamine and sulfadoxine resistance.

Amplicons from target sequences were generated using multiplexed nested PCR assays with indexed primers, which themselves contain specific tags (barcodes) consisting of individual 8-base indices specific to the sample and adapter sequences (14 or 15 bases) that allow the final PCR product to bind to the sequencing flow cell. A total of 4 µL of PCR reactions from each sample were mixed into a pool (96 samples). This was done to increase the sample volume and minimize the number of samples for downstream steps in the protocol. For each pool, amplicons were then purified with AMPure XP beads (Beckman Coulter) following the manufacturer’s protocol to remove dNTPs, salts, primers, and primer dimers. The quality of the purified PCR products was assessed by analysing the eluates containing the purified amplicons on a fragment analyzer (Agilent). The concentration of the DNA in the pooled fragments was assessed by fluorometric quantification (Qubit, Thermo Fisher). The pooled libraries were denatured with NaOH to a final concentration of 0.1 N and diluted with a hybridization buffer before sequencing. Sequences were performed with MiSeq v2 reagents using the 300-cycle kit (Illumina) as recommended by the manufacturer.

The raw sequences were demultiplexed and quality trimmed with a phred score of 30 to avoid primer bias. Primer sequences were trimmed from 5′ ends of sequences to avoid primer bias in sequenced fragments. Reads were compared to a custom database consisting of the 3D7 reference sequence for base calling. The bioinformatics analysis was carried out using the CLC Genomics Workbench 22 software package (Qiagen). Laboratory reference parasite strains (Dd2, 7G8, HB3, and Cambodian culture-adapted) with known alleles in each gene served as controls.

### Ethical considerations

The protocol was approved and received authorisation no. 2022-05-117 from the Burkina Faso Health Research Ethics Committee. Before the start of the study, a community meeting was held in each selected village to discuss the study with community leaders. Individual informed consent was obtained from each head of household during a home visit before any study procedure.

### Statistical analysis

The sociodemographic characteristics of the participants were described using summarising statistics. Continuous variables were described using mean (and standard deviation), median (and 95% confidence interval), and range. Continuous variables were compared using ANOVA. Categorical variables were presented as frequencies and proportions. Chi-squared or Fisher tests were used to assess differences in mutation and genotype rates between sites and individual parameters, including sex (female and male), age groups (6–59 months and 60 months to 10 years), parasite density groups (< 500 p/µL isolates, 501–5000 p/µL isolates, 501–50,000 p/µL isolates, and > 50,000 p/µL isolates) and body temperature groups (< 37.5 °C and ≥ 37.5 °C). A p-value test of < 0.05 was considered statistically significant. Analysis was carried out using MedCalc® Statistical Software version 20.218 (MedCalc Software Ltd, Ostend, Belgium; https://www.medcalc.org; 2023).

## Results

### Study population

A total of 1140 children were recruited across the three sites studied. However, only microscopically positive samples were used to extract parasite DNA from a total of 377 samples from the sites for the molecular marker of resistance study. The baseline characteristics of the study population by the site are summarized in Table 1. Significant differences in mean age and temperature were observed between the study populations at the 3 sites, as shown in Table 1.

**Table 1:** Baseline characteristics of the study individual by site

	Sites			All sites	p-value
	Banfora	Gaoua	Orodara		
No. of patients	75	244	58	377	–
Males, %	52%	48%	47%	49%	0.8
Age (years)					–
Mean (SD*)	5.7 (2.3)	5.60 (2.5)	6.7 (1.6)	5.8 (2.4)	< 0.001
Range (min–max)	0.6–10	0.5–10	2.2–9.6	0.5–10	–
Temperature (°C), day 0					–
Mean (SD*),	36.9 (0.5)	36.7 (0.6)	36.5 (0.4)	36.7 (0.6)	< 0.001
Parasitaemia (µl), day 0					–
Median (95% CI)	827 (509–1346)	940 (680–1240)	597 (276–1024)	800 (662–1059)	0.1
Range (min–max)	24–544,000	16–207,273	16–180,870	16–544,000	–

*SD* Standard deviation

### Mutations associated with resistance to anti-malarial drugs

Of the 377 samples analysed, 346 (91.7%), 369 (97.9%), 368 (97.6%), and 374 (99.2%) were successfully sequenced for *Pfcrt, Pfmdr-1, dhfr*, and *dhps*. There was no significant difference between the sites, except for *Pfcrt*, where the proportion of missing data was lower in Gaoua (3.7% in Gaoua vs. 18.6% and 16.0% in Banfora and Orodara, respectively, *p* < 0.0001).

### Mutations in *pfcrt* genes

*Pfcrt* mutations were observed at 5 codons (C72S, M74I, N75E, K76T, and I356T/L). The highest frequency was observed at codon 76T (9.8%), followed by M74I (9.5%), N75E (9.5%), and I356T (4.0%). The frequency of the other two non-synonymous mutations were below 1% (I356L, 0.9%, and C72S, 0.3%). *Pfcrt* mutations at codons 74, 75, 76, and 356 were significantly more frequent in Orodara (Table 2).

**Table 2:** Distribution of the *Pfcrt* mutations by site

Codons	Amino acid change	Site			All sites	p-value
		Banfora (%)	Gaoua (%)	Orodara (%)		
72	C > S	0.0	0.4	0.0	0.3	0.7
74	M > I	8.2	7.7	20.0	9.5	0.02
75	N > E	8.2	7.7	20.0	9.5	0.02
76	K > T	8.2	8.1	20.0	9.8	0.03
93	T > S	0.0	0.0	0.0	0.0	–
97	H > Y	0.0	0.0	0.0	0.0	–
145	F > I	0.0	0.0	0.0	0.0	–
218	I > F	0.0	0.0	0.0	0.0	–
343	M > L	0.0	0.0	0.0	0.0	–
350	C > R	0.0	0.0	0.0	0.0	–
353	G > V	0.0	0.0	0.0	0.0	–
356	I > T	4.9	2.1	12.0	4.0	0.02
	I > L	0.0	1.3	0.0	0.9	

A total of seven *Pfcrt* genotypes were identified. The wild-type genotype (CVMNKTHFIMCGI) predominated (89.3%), followed by the triple mutant (CV**IET**THFIMCGI, 5.8%) and the quadruple mutant (CV**IET**THFIMCG**T**, 3.5%). The remaining genotypes were found at frequencies below 1% (Table 3). The distribution of *Pfcrt* genotypes was similar between sites but differed significantly by sex (*p* = 0.035) with a 5-fold higher proportion of triple mutant genotype (CV**IET**THFIMCGI) in females than males (Table 3).

**Table 3:** Distribution of the seven *Pfcrt* genotypes detected by location

Genotype	Amino acid changes	N (%)	Sites			All site	p-value
			Banfora	Gaoua	Orodara		
CVMNKTHFIMCGI	Wild- type	N	55	214	40	309	0.16
		%	90.2%	91.1%	80.0%	89.3%	
CV**IET**THFIMCGI	74I/75E/76 T	N	3	13	4	20	
		%	4.9%	5.5%	8.0%	5.8%	
CV**IET**THFIMCG**T**	74I/75E/76 T/356 T	N	2	4	6	12	
		%	3.3%	1.7%	12.0%	3.5%	
CVMNKTHFIMCG**T**	356 T	N	1	1	0	2	
		%	1.6%	0.4%	0.0%	0.6%	
CV**IET**THFIMCG**L**	74I/75E/76 T/356L	N	0	1	0	1	
		%	0.0%	0.4%	0.0%	0.3%	
CVMNKTHFIMCG**L**	356L	N	0	1	0	1	
		%	0.0%	0.4%	0.0%	0.3%	
**S**VMN**T**THFIMCG**L**	72S/76 T/356L	N	0	1	0	1	
		%	0.0%	0.4%	0.0%	0.3%	

#### Mutations in *Pfmdr-1* genes

*Pfmdr-1* variants were observed at 4 codons (N86Y, Y184F, S1034C, and N1042D). Codon 184 F had the highest frequency (30.9%), followed by 86Y (1.9%). The proportion of the two other mutants were below 1% (1042D, 0.8%, and 1034 C, 0.3%). The distribution of the mutations was similar between the different sites (Table 4).

**Table 4:** Distribution of the *Pfmdr-1* mutations according to the site

Codons	Amino acid change	Sites			All sites (%)	p-value
		Banfora (%)	Gaoua (%)	Orodara (%)		
86	N > Y	1.4	2.5	0.0	1.9	0.4
184	Y > F	35.6	26.9	41.4	30.9	0.06
1034	S > C	0.0	0.4	0.0	0.3	0.7
1042	N > D	0.0	1.3	0.0	0.8	0.4
1246	D > Y	0.0	0.0	0.0	0.0	–

In total, six *Pfmdr-1* genotypes were found. The single mutant genotype (N**F**SND) predominated (66.7%), followed by the wild-type genotype (NYSND, 30.4%) and the double mutant genotype (**YF**SND, 1.4%). The 3 remaining genotypes were detected at frequencies less than 1% (Table 5). The distribution of *Pfmdr-1* genotypes was similar between sites. However, it differed significantly according to parasite density (p = 0.0002). The frequency of the single mutant genotype (N**F**SND) increased with increasing parasite density (49% for < 500 p/µL isolates, 75% for 501–5000 p/µL isolates, 81.8% for 501–50,000 p/µL isolates and 85% for > 50,000 p/µL isolates), while the frequency of the wild type (NYSND) decreased as parasite density increased (47.6% for < 500 p/µL isolates, 22.9% for 501–5000 p/µL isolates, 13.6% for 5001–50,000 p/µL isolates and 15.0% for > 50,000 p/µL isolates).

**Table 5:** Distribution of the seven *Pfmdr-1* genotypes detected according to their location

Genotype	Amino acid changes	N (%)	Sites			All site	p-value
			Banfora	Gaoua	Orodara		
NYSND	Whid- type	N	26	62	24	112	0.5
		%	35.6%	26.1%	41.4%	30.4%	
N**F**SND	184F	N	46	166	34	246	
		%	63.0%	69.7%	58.6%	66.7%	
**YF**SND	86Y/184F	N	1	4	0	5	
		%	1.4%	1.7%	0.0%	1.4%	
N**F**S**D**D	184F/1042D	N	0	3	0	3	
		%	0.0%	1.3%	0.0%	0.8%	
**Y**YSND	86Y	N	0	2	0	2	
		%	0.0%	0.8%	0.0%	0.5%	
N**FC**ND	184F/1034C	N	0	1	0	1	
		%	0.0%	0.4%	0.0%	0.3%	

#### **Mutations in the *dhfr *gene**

Mutations in the *dhfr* gene were observed at 3 codons (N51I, C59R, and S108N). The highest frequency was observed at codon 59R (69.8%). This was followed by codons 51I (66.6%) and 108 N (14.7%). The 108 N *dhfr* mutation was found to be significantly more frequent in Gaoua (Table 6).

**Table 6:** Distribution of the *dhfr* mutations by site

Codons	Amino acid change	Sites			All sites (%)	p-value
		Banfora (%)	Gaoua (%)	Orodara (%)		
16	A > V	0.0	0.0	0.0	0.0	–
50	C > R	0.0	0.0	0.0	0.0	–
51	N > I	60.8	68.2	67.2	66.6	0.5
59	C > R	70.3	69.0	72.4	69.8	0.9
108	S > N	6.8	19.0	6.9	14.7	0.006
164	I > L	0.0	0.0	0.0	0.0	–

A total of seven different *dhfr* genotypes were found (Table 7). The double mutant genotype (AC**IR**SI) predominated (52.4%). This was followed by the wild-type genotype (ACNCSI, 29.4%), the triple mutant genotype (AC**IRN**I, 13.6%), and the single mutant genotype (ACN**R**SI, 2.9%). The remaining 3 genotypes were detected at proportions below 1% (Table 7). The distribution of the *dhfr* genotypes differed significantly between the sites (p = 0.02): the double mutant genotype (AC**IR**SI) had a higher proportion in Orodora (62.1%) compared to Banfora (55.4%) and Gaoua (49. 2%), the triple mutant genotype (AC**IRN**I) was higher in Gaoua (18.2%) than in Banfora (5.4%) and Oradara (5.2%), and the single mutant genotype (ACNRSI) was higher in Banfora (8.1%) than in Oradara (3.4%) and Gaoua (1.2%). In addition, the distribution of the *dhfr* genotypes was significantly different according to the parasite density (*p* = 0.0001) and the axillary temperature (*p* = 0.01). The frequency of the triple mutant genotype (AC**IRN**I) increased with increasing parasite density (2.7% for < 500 p/µL isolates, 9.9% for 501-5000 p/µL isolates, 37.9% for 501–50,000 p/µL isolates and 40.0% for > 50,000 p/µL isolates), while the frequency of the wild-type (ACNCS) decreased as parasite density increased (45.9% for < 500 p/µL isolates, 26.8% for 501–5000 p/µL isolates, 7.6% for 5001–50,000 p/µL isolates and 0% for > 50,000 p/µL isolates). Similarly, the frequency of the triple mutant genotype (AC**IRN**I) increased with increasing body temperature (12.4% for isolates from individuals with < 37.5 °C and 25.0% for isolates from individuals with ≥ 37.5 °C), whereas the frequency of the wild type (ACNCSI) decreased with increasing body temperature (30.5% for isolates from individuals with < 37.5 °C and 19.4% for isolates from individuals with ≥ 37.5 °C).

**Table 7:** Distribution of the seven *dhfr* genotypes detected by site

Genotype	Amino acid changes	N (%)	Sites			All sites	p-value
			Banfora	Gaoua	Orodara		
ACNCSI	Wild type	N	22	72	16	110	0.02
		%	29.7%	29.8%	27.6%	29.4%	
AC**IR**SI	51II/59R	N	41	119	36	196	
		%	55.4%	49.2%	62.1%	52.4%	
AC**IRN**I	51I/59R/108N	N	4	44	3	51	
		%	5.4%	18.2%	5.2%	13.6%	
ACN**R**SI	59R	N	6	3	2	11	
		%	8.1%	1.2%	3.4%	2.9%	
ACN**RN**I	59R/108N	N	1	1	1	3	
		%	1.4%	0.4%	1.7%	0.8%	
AC**I**CSI	51I	N	0	2	0	2	
		%	0.0%	0.8%	0.0%	0.5%	
ACNC**N**I	108N	N	0	1	0	1	
		%	0.0%	0.4%	0.0%	0.3%	

#### **Mutations in the *dhps *genes**

Polymorphisms at codons 431, 436, 437, 540, 581, and 613 were observed. The highest frequency was observed at codon 437 K (89.3%). This was followed by codons 436 A (61.2%), 613 S (14.4%), and 431 V (1.1%). The other four mutations were below 1% (436 F 0.3%, 436Y 0.3%, 540E 0.3%, and 581G 0.8%). The 436 A *dhps* mutation was found to be significantly more frequent in Gaoua (Table 8).

**Table 8:** Distribution of the *dhps* mutations according to the site

Codons	Amino acid change	Sites			All sites (%)	p-value
		Banfora (%)	Gaoua (%)	Orodara (%)		
431	I > V	0.0	1.2	1.7	1.1	0.6
436	S > A	44.6	68.6	51.7	61.2	0.0008
	S > F	1.4	0.0	0.0	0.3	
	S > Y	1.4	0.0	0.0	0.3	
437	A > K	91.9	88.4	89.7	89.3	0.7
540	K > E	0.0	0.4	0.0	0.3	0.7
581	A > G	0.0	1.2	0.0	0.8	0.4
613	A > S	10.8	16.9	8.6	14.4	0.2

In total, 14 *dhps* genotypes were found (Table 9). The double mutant genotype (I**AK**KAA) and the single mutant genotype (IS**K**KAA) were predominant (37.7% and 37.2%, respectively). They were followed by the triple mutant genotype (I**AK**KA**S**, 12.3%), the single mutant genotype (I**A**AKAA, 8.8%), and the double mutant genotype (I**A**AKA**S**, 1.1%). The rest of the 9 genotypes had a frequency of less than 1% (Table 9). The distribution of *dhps* genotypes was similar between sites but differed significantly by parasite density (p = 0.006). The frequency of the single 436 A mutant genotype (I**A**AKAA) increased with increasing parasite density (6.2% at < 500 p/µL, 9.2% at 501 to 5000 p/µL, 12.1% at 501 to 50,000 p/µL, and 15. The frequency of the single 437 K mutant genotype (IS**K**KAA) decreased with increasing parasite density (54.1% at < 500 p/µL isolates, 27.5% at 501–5000 p/µL isolates, 27.3% at 5001–50,000 p/µL isolates and 15.0% at > 50 000 p/µL isolates).

**Table 9:** Distribution of the 14 *dhps* genotypes detected per location

Genotype	Amino acid changes	N (%)	Sites			All site	p-value
			Banfora	Gaoua	Orodara		
ISAKAA	Wild type	N	0	1	0	1	0.07
		%	0.0%	0.4%	0.0%	0.3%	
I**A**AKAA	436A	N	3	24	6	33	
		%	4.1%	9.9%	10.3%	8.8%	
I**F**AKAA	436F	N	1	0	0	1	
		%	1.4%	0.0%	0.0%	0.3%	
IS**K**KAA	437 K	N	39	72	28	139	
		%	52.7%	29.8%	48.3%	37.2%	
I**AK**KAA	436A/437 K	N	23	99	19	141	
		%	31.1%	40.9%	32.8%	37.7%	
I**A**AKA**S**	436A/613S	N	1	3	0	4	
		%	1.4%	1.2%	0.0%	1.1%	
I**Y**AKA**S**	436Y/613S	N	1	0	0	1	
		%	1.4%	0,0%	0.0%	0.3%	
IS**K**KA**S**	437 K/613S	N	0	2	0	2	
		%	0.0%	0.8%	0.0%	0.5%	
IS**K**K**G**A	437 K/581G	N	0	1	0	1	
		%	0.0%	0.4%	0.0%	0.3%	
I**AK**KA**S**	436A/437 K/613S	N	6	36	4	46	
		%	8.1%	14.9%	6.9%	12.3%	
I**AKE**AA	436A/437 K/540E	N	0	1	0	1	
		%	0.0%	0.4%	0.0%	0.3%	
**VAK**KAA	431 V/436A/437 K	N	0	1	0	1	
		%	0,0%	0.4%	0.0%	0.3%	
**VAK**K**G**A	431 V/436A/437 K/581G	N	0	2	0	2	
		%	0.0%	0.8%	0.0%	0.5%	
**VAK**KA**S**	431 V/436A/437 K/613S	N	0	0	1	1	
		%	0.0%	0.0%	1.7%	0.3%	

#### **Distribution of *dhfr/dhps *haplotypes**

The most frequent *dhfr/dhps* haplotypes were the triple mutant AC**IR**SI/I**A**KKAA (23%), followed by the wild-type ACNCSI/ISKKAA (19%) and the double mutant AC**IR**SI/ISKKAA (14%). The quintuple mutant haplotype N51I/C59R/S108N + G437A/540E (AC**IRN**I/IS**AE**AA) responsible for SP treatment failures in adults and children (but still effective for use in intermittent preventive treatment in pregnancy) was detected in one isolate from Gaoua. A septuple mutant AC**IRN**I/**VAK**K**G**A (triple mutant 51I/59R/108 N and quadruple mutant 431 V/436A/437K/581G) was observed in 2 isolates from Gaoua (0.5%).

## Discussion

This study was conducted to determine the current status of *P. falciparum* resistance to partner drugs in ACTs, as well as to SP, used alone for IPTp or in combination with amodiaquine for SMC [25, 33]. Parasites isolated from children aged 6 months to 10 years were tested for the prevalence of the known mutations *Pfcrt, Pfmdr-1, dhfr*, and *dhps*. The results showed a high prevalence of wild-type *Pfcrt* K76 (89.3%); similar to results from a study conducted in Burkina Faso five years after chloroquine discontinuation in 2010–2012 [7]. This confirms the results of several studies reporting chloroquine-susceptible strains circulating several years after CQ first-line treatment in Africa [7, 34, 35]. The triple mutant (CV**IET**THFIMCGI, 5.8%) and the quadruple mutant (CV**IET**THFIMCG**T**, 3.5%) were found at relatively low frequencies. The distribution of *Pfcrt* genotypes was similar between sites but was significantly higher for the triple mutant genotype (CV**IET**THFIMCGI) in females than in males. In Kenya, gender differences in the carriage of chloroquine-resistant genotypes have also been reported. This study however reported an opposite trend, males being more likely to harbor CQ-resistant *P. falciparum *[33]. The M74I and N75E mutations were present at a frequency of 9.5%, slightly less frequent than mutation at codon 76T (9.8%), previously observed at higher proportions in Burkina Faso [6, 7]. Here, 3.5% of the isolates tested had the I356T mutation, which has been associated with a decrease in quinine susceptibility and an increase in mefloquine susceptibility in African *P. falciparum *[36]. Additionally, one SVMNT mutant genotype was detected. SVMNT, mainly found in South America and Southeast Asia, is rare in Africa [37] and has been observed at a low frequency in Tanzania and Angola. It is thought that amodiaquine use may be a determining factor in the selection of the SVMNT haplotype in these regions [38, 39].

*Pfmdr-1* gene mutations such as the N86Y/Y184F/D1246Y have been associated to 4-aminoquinoline resistance and clinical failure to amodiaquine treatment [40]. In this study, the Y184F mutation was found to be the most common mutation, accounting for 66.7%, while the N86Y mutation was very rare (0.5%). No difference was found in the distribution of *Pfmdr-1* genotypes between sites. The double mutant genotype (**YF**SND) was detected at a frequency of 1.4% and the triple mutation was not found, consistent with recent results in symptomatic malaria patients described by Somé et al. [6]. Similar proportions were also reported in Nigeria and Senegal, where the 184 F allele was the more common allele [18, 41]. The low frequency of the 86Y allele corroborates the findings on recovering chloroquine-sensitive parasites. Mutation at the D1246Y locus which has been associated with quinine resistance [42], was not detected in this study. Mutations at 1034 C and 1042D, shown to be involved in the binding pocket of mefloquine, quinine, and chloroquine [19, 43], were observed at very low frequencies.

In Burkina Faso, only SP is used for IPTp or in combination with amodiaquine for SMC, two preventive strategies widely implemented in Sahelian countries. The study aimed to investigate the current impact of chemoprevention on the emergence and spread of SP-resistant malaria. Many studies have confirmed that the use of SP, whether for treatment or chemoprevention, is often associated with an increased prevalence of mutations in both *dhfr* and *dhps* genes [44–47]. For *dhfr*, the double mutant genotype (AC**IR**SI) predominated (52.4%), followed by the wild-type genotype (ACNCSI, 29.4%), the triple mutant genotype (AC**IRN**I, 13.6%) and the single mutant genotype (ACN**R**SI, 2.9%). No quadruple mutant (AC**IRNL**) was observed.

These findings support that the efficacy of IPTp-SP is not compromised in Burkina Faso. However, the distribution of the *dhfr* genotypes differed significantly between sites: a higher proportion of the double mutant genotype (AC**IR**SI) in Orodora (62.1%) and a higher proportion of the triple mutant genotype (AC**IRN**I) in Gaoua (18.2%). These data suggest that the pressure of the SP drug may be different depending on the location. Moreover, the higher proportion of triple mutants found in the location near the border with Ghana may indicate that parasites are circulating between the two countries. Indeed, a recent study conducted in Ghana indicated the presence of a high prevalence of triple mutations (AC**IRN**I) in the *dhfr* gene of *P. falciparum* isolates (71.4%) [48]. For mutations in the dhps gene, the most frequent mutations are located at codon 437 K, followed by codon 436 A. The codons 613 S, 431 V, 436 F 436Y, 540E, and 581G were also found at varying frequencies. The mutation A437G was not found as well in the study conducted in Niger on symptomatic malaria cases in pre-treatment [44]. The 436 A mutation has been described throughout Africa at varying levels of frequency [44–47]. Detecting the I431V mutation in this study, albeit at a low prevalence, confirms a previous finding from Ghana and Niger, two countries neighboring Burkina Faso [44, 45, 47]. The impact of the I431V mutation on the continued use of SP for IPTp remains to be elucidated [49]. A similar proportion of the A613S mutation (14.4%) was observed in this study compared with data reported from studies conducted in Ghana in children in 2017 [50]. The 581G and 613 S/T mutations have been detected at low prevalence in West and East Africa, but the rapid emergence of these mutations has been described in Kenya and Uganda [27, 51]. These mutations have been associated with high levels of resistance to sulfadoxine-pyrimethamine in East Africa [52]. Regarding the 540E mutation, our findings were also similar to those found in Ghana in pregnant women in antenatal care with a low frequency [47]. As these mutations may affect the use of SP for IPTp and SMC, it is important to continue to monitor the distribution and possible levels of these mutations [52].

Finally, the frequencies of the major mutants in the *Pfmdr-1* gene (N**F**SND) and in the *dhfr* gene (AC**IRN**I) increased with increasing parasite density, suggesting that drug resistance mutations can be selected for by exposure to large numbers of malaria parasites. It is also likely that individuals with high parasitaemia have limited immunity, particularly to the ‘strain’ causing infection, and that high parasitaemia is associated with a greater risk of selection for drug-resistant parasites [53].

## Conclusion

This study described the proportions of molecular markers *Pfcrt, Pfmdr-1, dhfr*, and *dhps* associated with resistance to SP and ACT partner drugs in asymptomatic malaria cases in high malaria transmission areas in Burkina Faso. Overall, a low proportion of mutants highly resistant to anti-malarial drugs was observed. Therefore, the monitoring of molecular markers of drug resistance should be continued. This will help to assess the dynamics of resistance and to select or modify the most effective treatment.

## Data Availability

All data generated or analysed during this study are available from the corresponding author and can be provided if required.
